# Hypothyroidism is associated with higher healthcare utilisation and higher need for blood transfusion after primary total knee arthroplasty (TKA) in patients with osteoarthritis: A National Inpatient Sample analysis

**DOI:** 10.1002/jeo2.70413

**Published:** 2026-01-30

**Authors:** Sumanth R. Chandrupatla, Kranti C. Rumalla, Jasvinder A. Singh

**Affiliations:** ^1^ Department of Medicine at the School of Medicine University of Alabama at Birmingham (UAB) Birmingham Alabama USA; ^2^ Feinberg School of Medicine, Northwestern University Chicago Illinois USA; ^3^ Medicine Service, Michael E. DeBakey VA Medical Center Houston Texas USA; ^4^ Department of Medicine Baylor College of Medicine Houston Texas USA

**Keywords:** hypothyroidism, knee arthroplasty, osteoarthritis of knee, treatment outcome

## Abstract

**Purpose:**

To examine the association of hypothyroidism with primary total knee arthroplasty (TKA) outcomes in adults with primary underlying diagnosis of osteoarthritis (OA).

**Methods:**

We identified a prospective cohort of patients in the 2016–2020 national inpatient sample (NIS) that received primary TKA with an underlying diagnosis of OA, identified using International Classification of Diseases, Tenth Revision, Common Modification (ICD‐10‐CM) and Procedure Coding System (ICD‐10‐PCS) codes in the primary procedure and diagnosis positions, respectively. We performed multivariable‐adjusted regression analyses for healthcare utilisation (length of hospital stay, hospital charges and discharge destination) and clinical outcomes (blood transfusion; prosthetic fracture, dislocation or infection; inpatient mortality), adjusted for age, sex, race, income, comorbidity, insurance payer, elective surgery, hospital bed size, census region and teaching status.

**Results:**

Between 2016 and 2020, we identified 2,922,075 adults who underwent primary TKA with an underlying diagnosis of OA, of whom 447,875 (16%) had hypothyroidism. For the primary TKA OA cohort, the average age was 66.8 years, 61.4% were female, and 81.3% were White. In the primary TKA OA cohort, hypothyroidism was associated with significantly higher multivariable adjusted odds ratio (aOR) for length of hospital stay above the median, 1.06 (95% confidence interval [CI]: 1.05–1.08, *p* < 0.001); total hospital charges above the median, aOR 1.07 (95% CI: 1.04–1.09, *p* < 0.001); non‐routine discharge, aOR 1.07 (95% CI: 1.05–1.09, *p* < 0.001); and the need for blood transfusion, aOR 1.15 (95% CI: 1.08–1.23, *p* < 0.001).

**Conclusion:**

Hypothyroidism was associated with increased healthcare utilisation and need for blood transfusion after primary TKA for OA. Future studies should investigate whether preoperative optimisation of hypothyroidism can positively improve primary TKA outcomes.

**Level of Evidence:**

Level II, prospective cohort study.

**Abbreviations**
AHRQ: Agency for Healthcare Research and Quality
aOR: adjusted odds ratio
CI: confidence interval
HCUP: Healthcare Cost and Utilisation Project
ICD‐10‐CM: International Classification of Diseases, Tenth Revision, Common Modification
ICD‐10‐PCS: International Classification of Diseases, Tenth Revision Procedure Coding System
ICF: intermediate care facility
LOS: length of in‐hospital stay
NIS: National Inpatient Sample
OA: osteoarthritis
PJI: peri‐prosthetic joint infection
PJI: peri‐prosthetic joint infection
SNF: Skilled Nursing Facility
THA: total hip arthroplasty
TKA: total knee arthroplasty

## INTRODUCTION

Knee osteoarthritis (OA) is one of the most common causes of disability in adults, with a significant proportion of adults aged 60 years and older showing evidence of knee OA [15, 24, 33], with limited treatment options. Primary total knee arthroplasty (TKA) an effective common procedure that leads to decreased pain, and improved function, quality of life and patient satisfaction [14, 32, 37]. TKA rates have been rising and are projected to increase significantly by 2040 [35, 36].

Hypothyroidism is a common condition [17, 23], with a prevalence between 0.3% and 3.7% in the United States depending on the population studied and definition used [9]. The prevalence of hypothyroidism is increasing in the United States, and its treatment success may be decreasing [45]. Whether hypothyroidism is associated with complications after TKA is unclear. Normal thyroid health is essential for the overall musculoskeletal system via T3 availability in osteoblasts and chondrocytes, affecting bone and cartilage repair [41, 42]. Hypothyroidism can potentially impact the immune system, and musculoskeletal function [3, 8, 19], including muscle mass and strength. Therefore, a pathophysiological link exists between thyroid function and bone and joint health, either directly or through the immune system.

The evidence for association of hypothyroidism with post‐TKA clinical outcomes is limited. Two single center studies found an association of hypothyroidism with increased 90‐day post‐TKA complications [21] and with prosthetic joint infection (PJI) after primary or revision total hip arthroplasty (THA) or TKA [39], and a 2005–2014 Medicare case–control TKA study reported that hypothyroidism was associated with 1.50‐times odds of PJI post‐TKA [7]. In contrast, a 2005–2014 US National Inpatient Sample (NIS) study found that hypothyroidism was protective against prothesis related complications of which PJI was the predominant complication [46]. In a single center study of TKA from 1993 to 1999, hypothyroidism was not associated with infection risk in multivariable analyses [30]. The contradictory evidence may be due to the differences in the sample (single center vs. national), age (Medicare vs. general US population), populations studied (mixed primary and revision THA/TKA vs. TKA), and study designs with differing rigour to address confounding bias and timeframe. Therefore, this question of whether hypothyroidism is associated with TKA clinical outcomes, including but not limited to peri‐prosthetic infection, fracture and dislocation, remains unanswered. Post‐arthroplasty complications are associated with higher healthcare utilisation and costs [47]. The introduction of bundled payments by US Medicare for joint replacement surgery more than a decade ago, has attracted attention to healthcare utilisation outcome, considered practical and important by all parties [1, 47].

Due to the rising rates of hypothyroidism and primary TKA, and the role of thyroid hormone in musculoskeletal health, we sought to investigate the association of hypothyroidism with primary TKA clinical and healthcare utilisation outcomes in people with osteoarthritis (OA), using a nationally representative from the US NIS [2]. We hypothesised that hypothyroidism will be associated with higher healthcare utilisation and worse clinical outcomes after primary TKA for OA.

## METHODS

### Data source

This study used data from the US NIS, provided by the Agency for Healthcare Research and Quality (AHRQ). The NIS is a 20% stratified nationally representative database of all inpatient hospital stays (excluding rehabilitation and long‐term acute care centres), regardless of payer in 48 US states, plus the District of Columbia [2]. The NIS can be weighted to create national estimates and is commonly used to create estimates of national healthcare utilisation and outcomes [2]. Beginning in the fourth quarter of 2015, and in the full year of 2016, the NIS began using the International Classification of Diseases, Tenth Revision, Common Modification (ICD‐10‐CM) and Procedure Coding System (ICD‐10‐PCS) coding system. Therefore, we only included the years 2016–2020 in our study to restrict to the period where coding was consistent, that is, ICD‐10. This study was approved by the Institutional Review Board at University of Alabama at Birmingham and adhered to the Declaration of Helsinki and to ethical principles of research. STROBE guidelines were followed (IRB‐120207004). No formal power analysis was performed, since this national dataset provides large sample size for all except rare outcomes.

### Study cohort

This NIS prospective cohort study included all patients whose primary diagnosis was OA that underwent primary TKA between 2016 and 2020, in whom we assessed outcomes during and after the procedure of interest. We selected these patients using ICD‐10‐PCS codes in the primary position for primary TKA, as follows: 0SRC*, 0SRD*, 0SRT*, 0SRU*, 0SRV and 0SRW* [20, 29].

Patients with OA using ICD‐10‐CM code M17 located in the primary diagnosis position were then selected. A diagnosis of hypothyroidism was determined based on the presence of one of the following ICD‐10‐CM codes in the secondary position, as previously: [12, 25] E02*, E03.0, E03.1, E03.8, and E03.9.

### Study outcomes and covariates

Our study focused on several key healthcare utilisation outcomes and clinical postoperative complications of primary TKA. All outcomes were pre‐specified. Healthcare utilisation outcomes of interest were length of in‐hospital stay (LOS), hospital charges, and non‐routine discharge. LOS and mean hospital charges were dichotomised as above or below the cohort median, due to a non‐normal distribution and for clinical interpretation, as in previous studies [11, 12]. Non‐routine discharge was defined as described by the NIS documentation as a discharge to a destination other than home, including discharge to a short‐term hospital, home health care, skilled nursing facility (SNF), intermediate care facility (ICF), or another type of facility, or patient leaving against medical advice [2].

Key clinical outcomes included inpatient mortality and postoperative complications. Postoperative complications were limited to only those that occurred as initial encounters during the index hospitalisation, or those coded as procedures and reported in the NIS: Need for blood transfusion; prosthetic fracture; prosthetic dislocation; and post‐procedural infection including Peri‐PJI. As evident in the documentation, many ICD‐10 codes do not specify any time relationships [10], due to the lack of specification of their occurrence during the current hospitalisation episode. Additionally, the NIS does not contain any additional present‐at‐admission indicators, and many previous studies have failed to adhere to the proper ICD‐10 and NIS guidelines [22, 27]. Therefore, adhering to these NIS guidelines, post‐arthroplasty complications were selected to only include those initial encounter diagnoses codes as to ensure that outcomes did not include any pre‐operative and/or chronic conditions, and using procedure codes for blood transfusions, which captures procedures during the index hospitalisation. All ICD‐10 codes used for postoperative complications are listed in Supporting Information: Table S1.

### Statistical analyses

Healthcare Cost and Utilisation Project (HCUP)‐provided estimates were used to weight the NIS and calculate the national utilisation rates of TKA for patients with hypothyroidism. The Cochran Armitage test was used to determine time trends between 2016 and 2019, excluding 2020 due to the COVID‐19 pandemic. Hospitalisations in the United States decreased by over 20% in 2020 due to the impact of COVID‐19 pandemic [28]. We used a chi squared test and a Wilcoxon test to determine trends in categorical and continuous outcomes, respectively.

Multivariable‐adjusted logistic regression analysis was used to determine if hypothyroidism was associated with healthcare utilisation and clinical postoperative complication outcomes of interest, using combined 2016–2020 NIS data. Analyses were adjusted for age, sex, race, Deyo–Charlson score, expected primary payer, median household income for ZIP code, hospital census region, hospital bed size, hospital control/ownership and elective versus non‐elective admission status.

Pre‐specified sensitivity analyses were performed to test the robustness of main study findings by additionally adjusting the main model for the following variables using sequential hierarchical models for: (1) emergency service indicator (indicating whether an admission utilised emergency services at a hospital); (2) All Patient Refined Diagnosis Related Groups (DRG) Severity of Illness Subclass (provides a numerical value which represents the severity of the diagnosis group); and (3) All Patient Refined DRG Risk of Mortality Subclass (provides a numerical value which represents the risk of mortality based on the diagnosis group). We used SPSS version 29 (Armonk, NY), and R 4.3.1 (Vienna, Austria) to perform these analyses.

## RESULTS

### Cohort characteristics and overall time‐trends

**Figure 1:**
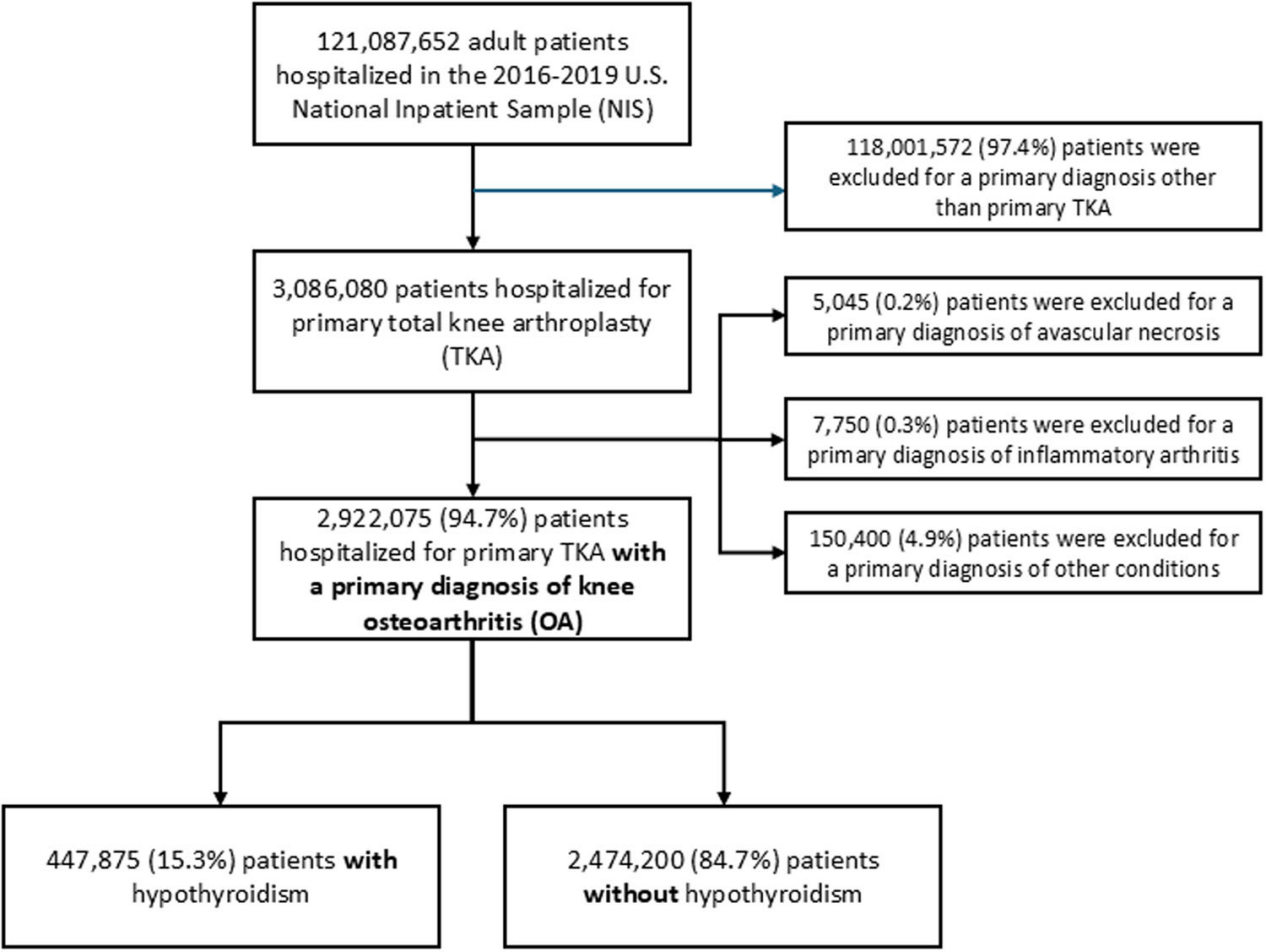
Study flow chart.

Between 2016 and 2020, 2,922,075 adults underwent primary TKA with an underlying diagnosis of OA, of whom 447,875 (15.3%) had hypothyroidism (Figure 1). For the entire primary TKA cohort, the average age was 66.8 years, 61.4% were female, 81.3% were White, and 43.3% had a Deyo–Charlson Comorbidity index ≥1 (Table 1).

**Table 1:** Characteristics of patients undergoing primary TKA 2016–2020, with a primary diagnosis of osteoarthritis, stratified by hypothyroidism.

	Patients with hypothyroidism (*N* = 447,875; 16%)	Patients without hypothyroidism (*N* = 2,444,200; 84%)	All patients (*N* = 2,922,075; 100%)
Age in years, mean (SD)	68.6 (9.1)	66.3 (9.5)	66.8 (9.4)
Sex, *N* (%)			
Male	88,085 (18.4)	1,038,390 (42.5)	1,126,475 (38.6)
Female	389,670 (81.6)	1,405,400 (57.5)	1,795,070 (61.4)
Race/ethnicity, *N* (%)			
White	397,430 (86.5)	1,888,445 (80.3)	2,285,875 (81.3)
Black	18.840 (4.1)	209,030 (8.9)	227,870 (8.1)
Hispanic	24,795 (5.4)	151,845 (6.5)	176,640 (6.3)
Asian or Pacific Islander	6105 (1.3)	38,135 (1.6)	44,240 (1.6)
Other	12.275 (2.7)	64,424 (2.7)	76,700 (2.7)
Deyo–Charlson Comorbidity Index, N (%)			
0	246,690 (51.6)	1,409,775 (57.7)	1,656,465 (56.7)
1	145,900 (30.5)	687,380 (28.1)	833,280 (28.5)
≥2	85,285 (17.8)	347,045 (14.2)	432,330 (14.8)
Insurance type, *N* (%)			
Medicare	310,615 (65.1)	1,350,970 (55.3)	1,661,585 (56.9)
Medicaid	14,505 (3.0)	110,985 (4.5)	125,490 (4.3)
Private insurance, self‐pay, no charge or other	152,255 (31.9)	979,145 (40.1)	1,131,400 (38.8)
Median Household Income for ZIP Code, *N* (%)			
0–25th percentile	98,225 (20.8)	536,915 (22.3)	635,140 (22.0)
26th–50th percentile	127,190 (27.0)	641,635 (26.6)	768,825 (26.7)
51st–75th percentile	129,090 (27.4)	651,625 (27.0)	780,715 (27.1)
76th–100th percentile	117,120 (24.8)	579,250 (24.0)	696,370 (24.2)
Hospital bed size, *N* (%)			
Small	153,755 (32.2)	799,850 (32.7)	953,605 (32.6)
Medium	135,815 (28.4)	687,754 (28.1)	823,569 (28.2)
Large	188,305 (39.4)	956,595 (39.1)	1,144,900 (39.2)
Census division of hospital, *N* (%)			
Northeast	87,745 (18.4)	471,931 (19.3)	559,676 (19.2)
Midwest	127,635 (26.7)	631,406 (25.8)	759,041 (26.0)
South	172,070 (36.0)	871,780 (35.7)	1,043,850 (35.7)
West	90,425 (18.9)	469,084 (19.2)	559,509 (19.1)
Location/teaching status of hospital, *N* (%)			
Rural	49,190 (10.3)	255,330 (10.4)	304,520 (10.4)
Urban non‐teaching	129,015 (27.0)	662,251 (27.1)	791,266 (27.1)
Urban teaching	299,670 (62.7)	1,526,619 (62.5)	1,826,289 (62.5)
Emergency service			
No emergency department (ED) indicator	474,845 (99.4)	2,426,050 (99.3)	2,900,895 (99.3)
Emergency department record	3030 (0.6)	18,150 (0.7)	21,180 (0.7)

Abbreviations: SD, standard deviation; TKA, total knee arthroplasty.

Compared to people without hypothyroidism, those with hypothyroidism undergoing primary TKA for OA were 2.3 years older (68.6 vs. 66.3), a higher percentage were female (81.6% vs. 57.5%), White (86.5% vs. 80.3%), or had a Deyo–Charlson Comorbidity Index ≥1 (48.3% vs. 42.3%), and a higher proportion had Medicare as their primary payer (65.1% vs. 55.3%) (Table 1). Between those undergoing primary TKA with OA and the entire primary TKA cohort, there was no significant differences, except that those with OA were younger (66.8 years vs. 68.7 years; Supporting Information: Table S2).

Unadjusted total hospital charges ($53,037 vs. $52,163), and rates of non‐routine discharge (65.3% vs. 61.2%) were numerically higher when comparing those with and without hypothyroidism (Table 2).

**Table 2:** Unadjusted differences in outcomes by hypothyroidism in people who underwent primary TKA for OA.

	Patients with hypothyroidism (*N* = 447,875; 16%)	Patients without hypothyroidism (*N* = 2,444,200; 84%)
Length of hospital stay in days, median (IQR)	2 (1–3)	2 (1–3)
Total hospital charges in $, median (IQR)	53,037.0 (38,524.5–74,951.0)	52,163.0 (37,970.0–74,409.0)
Inpatient mortality, *N* (%)	150 (0.03)	545 (0.02)
Non‐routine discharge, *N* (%)	311,725 (65.3)	1,495,285 (61.2)
Complications (initial visit), *N* (%)		
Need for blood transfusion	6745 (1.4)	25,365 (1.0)
Prosthetic fracture	595 (0.1)	2480 (0.1)
Prosthetic dislocation	85 (0.02)	465 (0.02)
Post‐procedural infection*	60 (0.01)	260 (0.01)
Peri‐prosthetic joint infection (PJI)	40 (0.01)	185 (0.01)

*Note*: Includes the presence of one or more of the following conditions based on the respective ICD‐10 codes: PJI, deep surgical site infection (SSI), and/or post‐procedural sepsis.

Abbreviations: IQR, interquartile range; OA, osteoarthritis; TKA, total knee arthroplasty.

In the overall TKA cohort with an underlying diagnosis of OA, between 2016 and 2019, a significant decrease in rates of non‐routine discharge (−8.1%; *p* < 0.001) and blood transfusion (−48.3%; *p* < 0.001; Supporting Information: Table S3) and significant increase in median hospital charges (5.5%; *p* < 0.001; Supporting Information: Table S3) were noted.

### Time trend analysis of primary TKA for OA utilisation rates and outcomes in hypothyroidism

A statistically significant increase in the proportion of primary TKA patients with OA as the underlying diagnosis who had hypothyroidism was noted between 2016 and 2019 (*p* < 0.001; Supporting Information: Table S4).

Between 2016 and 2019, the number of people undergoing primary TKA with an underlying diagnosis of OA decreased, while the number of people undergoing primary TKA for any diagnosis also decreased (Supporting Information: Table S4). There was a similar decrease in the total number of patients without hypothyroidism undergoing TKA, while the total number of patients with hypothyroidism decreased significantly less between 2016 and 2019 (Supporting Information: Figure S1). There was an expected sharp decrease in the OA and non‐hypothyroid cases in 2020, but hypothyroid numbers decreased less, as seen prior to 2020 (Supporting Information: Figure S1).

Between 2016 and 2019, in patients with hypothyroidism undergoing primary TKA for OA, there was a decrease in rates of non‐routine discharge, 69.1% vs. 63.3% (−8.4%, *p* < 0.001), and need for blood transfusion, 2.1% versus 1.1% (−46.6%, *p* < 0.001; Table 3). A significant increase in hospital charges, $51,310 versus $54,185, was also noted (5.6%, *p* < 0.001; Table 3). All these trends mirrored those in the primary TKA cohort with OA as the underlying diagnosis (Supporting Information: Table S3).

**Table 3:** Time‐trends in post‐primary TKA outcomes with an underlying diagnosis of OA in people with hypothyroidism from 2016 to 2020.

	All patients	Study time periods					Comparison of 2019–2016	
	2016–2020	2016	2017	2018	2019	2020	Last–first period (% difference)	*p* value
Length of hospital stay in days, median (IQR)	2 (1–3)	2 (2–3)	2 (2–3)	2 (1–3)	2 (1–3)	2 (1–2)	0	N/A
Total hospital charges in $, median (IQR)	53,037.0 (38,524.5–74,951)	51,310.0 (37,461.0–71,241.5)	51,280.0 (38,328.5–73,181.5)	53,929.0 (38,894.0–76,487.0)	54,185.0 (39,169.0–77,552.0)	55,934.0 (39,878.0–80,966.0)	**5.6**	**<0.001**
Inpatient mortality, *N* (%)	150 (0.03)	20 (0.02)	40 (0.03)	45 (0.04)	25 (0.03)	20 (0.04)	145.4	0.55
Non‐routine discharge, *N* (%)	330,924 (65.5)	86,910 (69.1)	83,975 (66.2)	68,575 (64.7)	60,145 (63.3)	31,320 (60.8)	**−8.4**	**<0.001**
Complications (initial visit), *N* (%)								
Need for blood transfusion	6745 (1.4)	2400 (2.1)	1630 (1.3)	1260 (1.2)	990 (1.1)	465 (1.0)	**−46.6**	**<0.001**
Prosthetic fracture	595 (0.1)	155 (0.1)	195 (0.2)	100 (0.1)	85 (0.1)	60 (0.1)	−29.0	0.29
Prosthetic dislocation	85 (0.02)	15 (0.01)	10 (0.01)	15 (0.01)	20 (0.02)	25 (0.05)	72.5	0.08
Post‐procedural infection*	60 (0.01)	15 (0.01)	**	20 (0.02)	**	**	**	N/A
Peri‐prosthetic joint infection	40 (0.01)	15 (0.01)	**	**	**	**	**	N/A

*Note*: N/A, could not be assessed since there was no difference between 2016 to 2019, or cell size was too small or missing to perform a test of statistical significance. Significant *p*‐values are bolded.

Abbreviations: HCUP, Healthcare Cost and Utilization Project; ICD‐10, International Classification of Diseases, Tenth Revision; IQR, interquartile range; OA, osteoarthritis; TKA, total knee arthroplasty.

*Includes the presence of one or more of the following conditions based on the respective ICD‐10 codes: periprosthetic joint infection (PJI), deep surgical site infection (SSI), and/or post‐procedural sepsis

**Data not presented as per the HCUP guidelines; for cells with counts <20 are not to be presented.

### Multivariable‐adjusted association of hypothyroidism with primary TKA outcomes

Hypothyroidism was significantly associated with length of hospital stay above the median, total hospital charges above the median, non‐routine discharge, and blood transfusion (Table 4; Supporting Information: Figure S2). No associations were noted for inpatient mortality or other postoperative complications (Table 4). Additional sensitivity analysis that controlled for emergency service indicator, and All Patient Refined DRG Severity of Illness Subclass, and All Patient Refined DRG Risk of Mortality Subclass, replicated the main findings (Table 5).

**Table 4:** Multivariable‐adjusted** association of hypothyroidism with post‐primary TKA outcomes from NIS 2016–2020, overall and by the underlying diagnosis.

	Osteoarthritis (*N* = 2,877,320)	
	aOR (95% CI)	*p* value
Length of hospital stay above the median [>2 days]	**1.06 (1.05–1.08)**	**<0.001**
Total hospital charges above the median [>$52,308]	**1.07 (1.04–1.09)**	**<0.001**
Inpatient mortality	1.39 (0.91–2.11)	0.13
Non‐routine discharge	**1.07 (1.05–1.09)**	**<0.001**
Complications		
Need for blood transfusion	**1.15 (1.08–1.23)**	**<0.001**
Prosthetic fracture	1.01 (0.82–1.24)	0.94
Prosthetic dislocation	0.85 (0.50–1.44)	0.54
Post‐procedural infection*	1.36 (0.71–2.62)	0.35
Periprosthetic joint infection (PJI)	1.23 (0.55–2.76)	0.62

*Note*: Significant *p*‐values and adjusted odds ratios (aOR) are bolded.

Abbreviations: CI, confidence interval; NIS, National Inpatient Sample; TKA, total knee arthroplasty.

*Includes periprosthetic joint infection (PJI), deep surgical site infection (SSI), and post‐procedural sepsis.

**Multivariable‐adjusted model includes age, sex, census division of hospital, race, hospital teaching status, median household income for ZIP code, expected primary payer, Deyo–Charlson score, hospital bed size, hospital control/ownership, and elective versus non‐elective admission

**Table 5:** Sensitivity analyses: Multivariable‐adjusted** association of hypothyroidism with post‐primary TKA outcomes from NIS 2016–2020, after adding the following to the main model, emergency service indicator, All Patient Refined DRG: Severity of Illness Subclass and All Patient Refined DRG: Severity of Illness and Risk of Mortality Subclasses.

	Sensitivity analysis 1: Adjusting for emergency service indicator^a^		Sensitivity analysis 2: Adjusting for emergency service indicator and All Patient Refined DRG: Severity of Illness Subclass^b^		Sensitivity analysis 3: Adjusting for emergency service indicator and All Patient Refined DRG: Severity of Illness and Risk of Mortality Subclasses^c^	
	aOR (95% CI)	*p* value	aOR (95% CI)	*p* value	aOR (95% CI)	*p* value
Length of hospital stay above the median [>2 days])	**1.06 (1.05–1.08)**	**<0.001**	**1.04 (1.02–1.05)**	**<0.001**	**1.03 (1.01–1.05)**	**<0.001**
Total hospital charges above the median [>$52,308]	**1.07 (1.04–1.09)**	**<0.001**	**1.06 (1.03–1.08)**	**<0.001**	**1.05 (1.03–1.08)**	**<0.001**
Inpatient mortality	1.39 (0.91–2.11)	0.13	1.37 (0.89–2.10)	0.15	1.35 (0.88–2.07)	0.18
Non‐routine discharge	**1.07 (1.05–1.09)**	**<0.001**	**1.06 (1.04–1.08)**	**<0.001**	**1.06 (1.04–1.08)**	**<0.001**
Complications						
Need for blood transfusion	**1.15 (1.08–1.23)**	**<0.001**	**1.08 (1.01–1.15)**	**0.03**	**1.07 (1.00–1.14)**	**0.04**
Prosthetic fracture	1.01 (0.82–1.25)	0.93	0.98 (0.80–1.21)	0.88	0.99 (0.80–1.22)	0.89
Prosthetic dislocation	0.85 (0.50–1.44)	0.54	0.81 (0.48–1.37)	0.43	0.81 (0.48–1.37)	0.43
Post‐procedural infection*	1.36 (0.71–2.62)	0.35	1.25 (0.65–2.39)	0.50	1.23 (0.64–2.37)	0.53
Periprosthetic Joint infection	1.23 (0.55–2.77)	0.61	1.13 (0.51–2.52)	0.77	1.12 (0.50–2.49)	0.79

Abbreviations: aOR, adjusted odds ratio; CI, confidence interval; NIS, National Inpatient Sample; TKA, total knee arthroplasty.

* Includes periprosthetic joint infection (PJI), deep surgical site infection (SSI), and post‐procedural sepsis.

** All multivariable‐adjusted models include age, sex, census division of hospital, race, hospital teaching status, median household income for ZIP code, expected primary payer, Deyo–Charlson score, hospital bed size, hospital control/ownership, and elective versus non‐elective admission.

^a^ Emergency service indicator—indicates whether an admission utilised the hospital's emergency services.

^b^ All Patient Refined Diagnosis Related Groups (DRG): Severity of Illness Subclass—Measures the severity of a patient's DRG.

^c^ All Patient Refined DRG: Risk of Mortality Subclasses—Measures the risk of mortality for a patient based on their assigned DRG.

## DISCUSSION

In this national sample of primary TKA for OA, hypothyroidism was independently associated with higher odds of a longer length of hospital stay, higher hospital charges, non‐routine discharge, and blood transfusion, after adjusting for patient and hospital factors. Suboptimal thyroid replacement therapy for hypothyroidism might impact bone healing [41, 42], in some patients, at least partially. Hypothyroidism is associated with reduced muscle mass and strength [3, 8, 19], which may be related to the effect of thyroid hormone on glucose uptake in muscle and adipose tissue and suppression of lipolysis [16]. Hypothyroidism associated myopathy (in severe cases) can interfere with post‐TKA recovery, rehabilitation and independence, which can lead to higher healthcare utilisation. Thus, hypothyroidism associated reduced muscle mass and strength, might contribute to a slower post‐TKA recovery, further leading to longer hospital stays, non‐home discharge and higher resource use by these patients. These observations are clinically important and relevant. Our findings add to the current knowledge. In a Medicare age‐sex‐comorbidity‐matched case–control study, hypothyroidism was associated with higher 90‐day care charges after primary TKA [7]. In a single center age‐sex‐matched analysis, hypothyroidism was associated with higher rates of non‐home discharge in people who underwent primary TKA [21]. Our study extends these findings from Medicare‐eligible elderly population and a single site experience to a nationally representative sample of Americans, using a robust analytic approach that controlled for demographic, clinical, comorbidity, income, hospital, and payer characteristics. Findings should be generalisable to all Americans who undergo primary TKA for OA, since U.S. NIS is a representative national sample.

Our study identifies hypothyroidism as a risk factor for more blood transfusions after primary TKA, a suboptimal clinical outcome. One of the reasons for the association of hypothyroidism with a higher need for blood transfusion is hypothyroidism associated baseline preoperative anaemia [43], which might be due to decreased erythropoietin production, bone marrow suppression, or concomitant nutritional deficiency (iron, vitamin B12 or folate) [38]. Additional proposed mechanisms include hypothyroidism associated increased perioperative blood loss, leading to postoperative anaemia [40]. Both mechanisms can contribute to higher blood transfusion need. No associations were noted for peri‐prosthetic fracture, dislocation and infection, and inpatient mortality, which are important negative study findings.

Thyroid replacement therapy for hypothyroidism is easily accessible and effective. Preoperative optimisation of hypothyroidism prior to primary TKA should be a reasonable medical management goal [4, 5], since primary TKA for OA is an elective procedure. Optimisation of thyroid replacement therapy has the potential to improve post‐TKA clinical outcomes and potentially reduce complications, which needs further study with a prospective, randomised trial.

Our study findings add to the current knowledge regarding factors associated with post‐TKA complications. Previous studies that examined the relationship between hypothyroidism and post‐primary TKA outcomes were limited to [1] single center, non‐nationally representative studies [2, 21, 30, 39], included only Medicare‐eligible population [3, 7] combined primary TKA with other arthroplasty populations [39], or [4] used ICD‐9 coding for the case definition [7]. The NIS lacks a present‐on‐admission indicator to differentiate historic from new diagnoses. In contrast to the ICD‐10 codes, the ICD‐9 codes do not have “initial visit” indicator, so the use of ICD‐9 codes for secondary diagnoses from the NIS in previous studies means that pre‐operative diagnoses/complications could be erroneously included as index visit complications. The 2016–2020 period only includes ICD‐10 codes in the US NIS. We only included postoperative complications coded as initial encounter in this study, which meant that these were ongoing active diagnoses during the index primary TKA hospitalisation, or the first visit for these complications. This approach likely led to a greater certainty for complications being acute and occurring during the index hospitalisation.

In the primary TKA population with underlying diagnosis of OA, 16% had hypothyroidism, similar to the previously reported prevalence in this cohort of people [6]. The prevalence of hypothyroidism is between 0.3% and 3.7% in the United States depending on the population studied and definition used [9], although others have reported a higher prevalence between 4% and 11% [45]. Our observation of a higher prevalence of hypothyroidism in primary TKA versus the national estimates, may be at least partially be related to: (1) an older age of TKA recipients (mean age, 65–68 years) versus the general US population; (2) the association of hypothyroidism with obesity, a strong risk factor for primary TKA [34, 44]; and (3) the association of hypothyroidism with OA and with osteoclast and osteoblast function [18, 41, 42].

We noted a decrease in the number of primary TKAs among patients with OA occurring in US hospitals from 2018 to 2020, slightly different than the overall primary TKA numbers that continue to increase year‐over‐year, except an expected drop during the COVID‐19 pandemic. This change was seen in the non‐hypothyroid population, and the magnitude was much smaller in the hypothyroid population. Prior to 1 January 2018, primary TKA was a part of the Centres for Medicare and Medicaid Services Inpatient‐Only list, meaning that all primary TKA surgeries had to be done in inpatient setting [26]. After 1/1/2018, Medicare primary TKA was permitted in outpatient settings. Due to this change, there has been a noticeable decrease in the number of inpatient primary TKA cases and a corresponding rise in the number of outpatient primary TKA cases, which constitutes up to 40% of all TKA cases as of 2020 according to some estimates [13, 31]. An effect of this evolving practice was also seen in our study, with a decrease in primary TKA annual utilisation beginning in 2018 among the overall OA cohort, and sub‐cohorts.

The study findings need to be reviewed in the context of certain limitations. Since we used diagnostic codes to identify hypothyroidism, under‐ or over‐reporting are possible, that is, misclassification bias should be considered. Misclassification bias would bias our estimate towards the null, making our estimates conservative. The NIS also does not include federal institutions (Veterans Affairs and military hospitals), indicating these findings may not be generalisable to this subpopulation. The NIS does not contain any longitudinal data, thus limiting our ability to examine long‐term outcomes. NIS does not include body mass index (BMI), or laboratory data (i.e., thyroid hormone levels), important factors that were most desirable to be studied and adjusted in the analysis. Finally, since our study included patient data from 2020, interpretation of the data needs to consider the impact of the COVID‐19 pandemic on patient outcomes and trends. BMI data are not available in the US NIS, and therefore we were unable to adjust for this important potential confounding variable.

Our study also has various strengths. Our use of nationally representative data allows these findings to be generalisable to the US population. We used the most contemporary years of the NIS, 2016–2020, that used ICD‐10 codes, which allowed for a greater precision in assessing postoperative complications. The NIS does not contain a present‐at‐admission indicator. We selected postoperative complication outcomes that were coded as a procedure (blood transfusion), or as initial visits (prosthetic fracture, prosthetic dislocation, or PJI (deep SSI or sepsis]) to prevent the inclusion of historic diagnoses.

## CONCLUSION

In conclusion, we identified an association of hypothyroidism higher healthcare utilisation and the need for blood transfusion, in patients undergoing primary TKA for OA. Hypothyroidism was associated with increased odds of a longer hospital stay, higher hospital charges, non‐routine discharge, and the need for blood transfusion. No associations were noted for inpatient mortality or postoperative complications including prosthetic fracture, dislocation or infection. Future studies should examine whether targeted pre‐operative therapy to optimise thyroid function can reduce healthcare utilisation and improve clinical outcomes post‐primary TKA for people with OA as the underlying diagnosis.

## AUTHOR CONTRIBUTIONS

Sumanth Chandrupatla and Jasvinder A. Singh designed the study, and developed the study protocol. Sumanth Chandrupatla accessed the data and performed the analyses, under the supervision of Jasvinder A. Singh. Sumanth Chandrupatla and Jasvinder A. Singh wrote the first draft of the paper. All authors reviewed analyses, revised the manuscript, read, and approved the final manuscript.

## CONFLICT OF INTEREST STATEMENT

JAS has received consultant fees from ROMTech, Atheneum, Clearview healthcare partners, American College of Rheumatology, Yale, Hulio, Horizon Pharmaceuticals/DINORA, ANI/Exeltis, USA Inc., Frictionless Solutions, Schipher, Crealta/Horizon, Medisys, Fidia, PK Med, Two labs Inc., Adept Field Solutions, Clinical Care options, Putnam associates, Focus forward, Navigant consulting, Spherix, MedIQ, Jupiter Life Science, UBM LLC, Trio Health, Medscape, WebMD, and Practice Point communications; the National Institutes of Health; and the American College of Rheumatology. JAS has received institutional research support from Zimmer Biomet Holdings. JAS received food and beverage payments from Intuitive Surgical Inc./Philips Electronics North America. JAS owns stock options in Atai life sciences, Kintara therapeutics, Intelligent Biosolutions, Acumen pharmaceutical, TPT Global Tech, Vaxart pharmaceuticals, Atyu biopharma, Adaptimmune Therapeutics, GeoVax Labs, Pieris Pharmaceuticals, Enzolytics Inc., Seres Therapeutics, Tonix Pharmaceuticals Holding Corp., Aebona Pharmaceuticals, and Charlotte's Web Holdings, Inc. JAS previously owned stock options in Amarin, Viking and Moderna pharmaceuticals. JAS is on the speaker's bureau of Simply Speaking. JAS was a member of the executive of Outcomes Measures in Rheumatology (OMERACT), an organisation that develops outcome measures in rheumatology and receives arms‐length funding from 8 companies. JAS serves on the FDA Arthritis Advisory Committee. JAS is the co‐chair of the Veterans Affairs Rheumatology Field Advisory Board (FAB). JAS is the editor and the Director of the University of Alabama at Birmingham (UAB) Cochrane Musculoskeletal Group Satellite Center on Network Meta‐analysis. JAS previously served as a member of the following committees: member, the American College of Rheumatology's (ACR) Annual Meeting Planning Committee (AMPC) and Quality of Care Committees, the Chair of the ACR Meet‐the‐Professor, Workshop and Study Group Subcommittee and the co‐chair of the ACR Criteria and Response Criteria subcommittee. All other authors declare no relevant conflict of interest.

## ETHICS STATEMENT

This study was approved by the Institutional Review Board at University of Alabama at Birmingham and adhered to the Declaration of Helsinki and to ethical principles of research. STROBE guidelines were followed (IRB‐120207004).

## Supporting information

Supporting information.

## Data Availability

All data are provided by the Healthcare Cost and Utilisation Project (HCUP), Agency for Healthcare Research and Quality, and is publicly available at https://hcup-us.ahrq.gov/.

## References

[1] Agarwal R , Liao JM , Gupta A , Navathe AS . The impact of bundled payment on health care spending, utilization, and quality: a systematic review. Health Aff. 2020;39:50–57. 10.1377/hlthaff.2019.00784 31905061

[2] Agency for Healthcare Research and Quality . HCUP National Inpatient Sample (NIS). Healthcare Cost and Utilization Project (HCUP). 2020. Available from: www.hcup-us.ahrq.gov/nisoverview.jsp

[3] Antonelli A , Delle Sedie A , Fallahi P , Ferrari SM , Maccheroni M , Ferrannini E , et al. High prevalence of thyroid autoimmunity and hypothyroidism in patients with psoriatic arthritis. J Rheumatol. 2006;33:2026–2028. 17014017

[4] Bekkering GE , Agoritsas T , Lytvyn L , Heen AF , Feller M , Moutzouri E , et al. Thyroid hormones treatment for subclinical hypothyroidism: a clinical practice guideline. BMJ. 2019;365:l2006. 10.1136/bmj.l2006 31088853

[5] Biondi B , Cooper DS . Thyroid hormone therapy for hypothyroidism. Endocrine. 2019;66:18–26. 10.1007/s12020-019-02023-7 31372822

[6] Bozic KJ , Lau E , Kurtz S , Ong K , Berry DJ . Patient‐related risk factors for postoperative mortality and periprosthetic joint infection in medicare patients undergoing TKA. Clin Orthop Relat Res. 2012;470:130–137. 10.1007/s11999-011-2043-3 PMC3237966 21874391

[7] Buller LT , Rosas S , Sabeh KG , Roche MW , McLawhorn AS , Barsoum WK . Hypothyroidism increases 90‐day complications and costs following primary total knee arthroplasty. J Arthroplasty. 2018;33:1003–1007. 10.1016/j.arth.2017.10.053 PMC6383647 29174407

[8] Cakir M , Samanci N , Balci N , Balci MK . Musculoskeletal manifestations in patients with thyroid disease. Clin Endocrinol. 2003;59:162–167. 10.1046/j.1365-2265.2003.01786.x 12864792

[9] Canaris GJ , Manowitz NR , Mayor G , Ridgway EC . The Colorado thyroid disease prevalence study. Arch Intern Med. 2000;160:526–534. 10.1001/archinte.160.4.526 10695693

[10] Centers for Disease Control and Prevention . International Classification of Diseases, (ICD‐10‐CM/PCS) Transition – Background. 2015. Available from: https://www.cdc.gov/nchs/icd/icd10cm_pcs_background.htm

[11] Chandrupatla S , Rumalla K , Singh JA . Association between diabetes mellitus and total hip arthroplasty outcomes: an observational study using the US National Inpatient Sample. BMJ Open. 2024;14:e085400. 10.1136/bmjopen-2024-085400 PMC11404163 39038867

[12] Chandrupatla SR , Rumalla KC , Singh JA . Hypothyroidism impacts clinical and healthcare utilization outcomes after primary total hip arthroplasty. J Arthroplasty. 2024;39:S279–S286.e3. 10.1016/j.arth.2023.11.005 37972668

[13] DeMik DE , Carender CN , An Q , Callaghan JJ , Brown TS , Bedard NA . Has removal from the inpatient‐only list increased complications after outpatient total knee arthroplasty? J Arthroplasty. 2021;36:2297–2301.e1. 10.1016/j.arth.2021.02.049 33714634

[14] DeRogatis M , Anis HK , Sodhi N , O. Ehiorobo J , Chughtai M , Bhave A , et al. Non‐operative treatment options for knee osteoarthritis. Ann Transl Med. 2019;7:S245. 10.21037/atm.2019.06.68 PMC6828999 31728369

[15] Dillon CF , Rasch EK , Gu Q , Hirsch R . Prevalence of knee osteoarthritis in the United States: arthritis data from the Third National Health and Nutrition Examination Survey 1991‐94. J Rheumatol. 2006;33:2271–2279. 17013996

[16] Dimitriadis G , Mitrou P , Lambadiari V , Boutati E , Maratou E , Panagiotakos DB , et al. Insulin action in adipose tissue and muscle in hypothyroidism. J Clin Endocrinol Metab. 2006;91:4930–4937. 10.1210/jc.2006-0478 17003097

[17] Escobar‐Morreale HF , Botella‐Carretero JI , del Rey FE , de Escobar GM . Treatment of hypothyroidism with combinations of levothyroxine plus liothyronine. J Clin Endocrinol Metab. 2005;90:4946–4954. 10.1210/jc.2005-0184 15928247

[18] Hellevik AI , Johnsen MB , Langhammer A , Fenstad AM , Furnes O , Storheim K , et al. Incidence of total hip or knee replacement due to osteoarthritis in relation to thyroid function: a prospective cohort study (The Nord‐Trøndelag Health Study). BMC Musculoskelet Disord. 2017;18:201. 10.1186/s12891-017-1565-6 PMC5437592 28521834

[19] Huang CM , Sung FC , Chen HJ , Lin CC , Lin CL , Huang PH . Hypothyroidism risk associated with rheumatoid arthritis: a population‐based retrospective cohort study. Medicine. 2022;101:e28487. 10.1097/MD.0000000000028487 PMC8735800 35029902

[20] Hung TY , Liu KL , Wen SH . Using the phecode system to identify the preoperative clinical phenotypes associated with surgical site infection in patients undergoing primary total knee arthroplasty: the sex differences. J Clin Med. 2022;11:5784. 10.3390/jcm11195784 PMC9573756 36233652

[21] Jing W , Long G , Yan Z , Ping Y , Mingsheng T . Subclinical hypothyroidism affects postoperative outcome of patients undergoing total knee arthroplasty. Orthop Surg. 2021;13:932–941. 10.1111/os.12934 PMC8126938 33817980

[22] Khera R , Angraal S , Couch T , Welsh JW , Nallamothu BK , Girotra S , et al. Adherence to methodological standards in research using the National Inpatient Sample. JAMA. 2017;318:2011–2018. 10.1001/jama.2017.17653 PMC5742631 29183077

[23] Kocełak P , Mossakowska M , Puzianowska‐Kuźnicka M , Sworczak K , Wyszomirski A , Handzlik G , et al. Prevalence and risk factors of untreated thyroid dysfunctions in the older Caucasian adults: results of PolSenior 2 survey. PLoS One. 2022;17:e0272045. 10.1371/journal.pone.0272045 PMC9394816 35994462

[24] Lee JK , Choi CH . Total knee arthroplasty in rheumatoid arthritis. Knee Surg Relat Res. 2012;24:1–6. 10.5792/ksrr.2012.24.1.1 PMC3341816 22570845

[25] Lin JY , Kao PC , Tsai YT , Chung CH , Chien WC , Lin CY , et al. Hypothyroidism is correlated with ventilator complications and longer hospital days after coronary artery bypass grafting surgery in a relatively young population: a nationwide, population‐based study. J Clin Med. 2022;11:3881. 10.3390/jcm11133881 PMC9267490 35807168

[26] MLN Matters . Total knee arthroplasty (TKA) removal from the Medicare Inpatient‐Only (IPO) list and application of the 2‐Midnight Rule. 2018. Available from: https://www.cms.gov/files/document/se18018pdf

[27] Mori M , Brown KJ , Geirsson A . Understanding limitations of the National Inpatient Sample to facilitate its proper use. JAMA Surgery. 2019;154:881–882. 10.1001/jamasurg.2019.1172 31141132

[28] Nguyen JL , Benigno M , Malhotra D , Khan F , Angulo FJ , Hammond J , et al. Pandemic‐related declines in hospitalization for non‐COVID‐19‐related illness in the United States from January through July 2020. PLoS One. 2022;17:e0262347. 10.1371/journal.pone.0262347 PMC8735608 34990489

[29] Ojemolon PE , Shaka H , Edigin E , Gomez TMA , Eseaton P , Bello J , et al. Impact of diabetes mellitus on outcomes of patients with knee osteoarthritis who underwent knee arthroplasty: an analysis of the Nationwide Inpatient Sample. Cureus 12:e8902. 2020. 10.7759/cureus.8902 PMC7389248 32742869

[30] Peersman G , Laskin R , Davis J , Peterson M . Infection in total knee replacement: a retrospective review of 6489 total knee replacements. Clin Orthop Relat Res. 2001;392:15–23. 11716377

[31] Rullán PJ , Xu JR , Emara AK , Molloy RM , Krebs VE , Mont MA , et al. Major national shifts to outpatient total knee arthroplasties in the United States: a 10‐year trends analysis of procedure volumes, complications, and healthcare utilizations (2010 to 2020). J Arthroplasty. 2023;38:1209–1216.e5. 10.1016/j.arth.2023.01.019 36693513

[32] Shan L , Shan B , Suzuki A , Nouh F , Saxena A . Intermediate and long‐term quality of life after total knee replacement: a systematic review and meta‐analysis. J Bone Jt Surg. 2015;97:156–168. 10.2106/JBJS.M.00372 25609443

[33] Sharma L . Osteoarthritis of the knee. N Engl J Med. 2021;384:51–59. 10.1056/NEJMcp1903768 33406330

[34] Singer SP , Dammerer D , Krismer M , Liebensteiner MC . Maximum lifetime body mass index is the appropriate predictor of knee and hip osteoarthritis. Arch Orthop Trauma Surg. 2018;138:99–103. 10.1007/s00402-017-2825-5 PMC5754409 29079909

[35] Singh JA , Yu S , Chen L , Cleveland JD . Rates of total joint replacement in the United States: future projections to 2020‐2040 using the National Inpatient Sample. J Rheumatol. 2019;46:1134–1140. 10.3899/jrheum.170990 30988126

[36] Sloan M , Premkumar A , Sheth NP . Projected volume of primary total joint arthroplasty in the U.S., 2014 to 2030. J Bone Jt Surg. 2018;100:1455–1460. 10.2106/JBJS.17.01617 30180053

[37] Steinhaus ME , Christ AB , Cross MB . Total knee arthroplasty for knee osteoarthritis: support for a foregone conclusion? HSS J. 2017;13:207–210. 10.1007/s11420-017-9558-4 PMC5481268 28690473

[38] Szczepanek‐Parulska E , Hernik A , Ruchala M . Anemia in thyroid diseases. Pol Arch Intern Med 127:352–360. 2017. 10.20452/pamw.3985 28400547

[39] Tan TL , Rajeswaran H , Haddad S , Shahi A , Parvizi J . Increased risk of periprosthetic joint infections in patients with hypothyroidism undergoing total joint arthroplasty. J Arthroplasty. 2016;31:868–871. 10.1016/j.arth.2015.10.028 26777546

[40] Tsotsolis S , Kenanidis E , Pegios VF , Potoupnis M , Tsiridis E . Is thyroid disease associated with post‐operative complications after total joint arthroplasty? A systematic review of the literature. EFORT Open Rev. 2023;8:54–62. 10.1530/EOR-22-0085 PMC9969002 36805936

[41] Waung JA , Bassett JHD , Williams GR . Thyroid hormone metabolism in skeletal development and adult bone maintenance. Trends Endocrinol Metab. 2012;23:155–162. 10.1016/j.tem.2011.11.002 22169753

[42] Williams GR . Thyroid hormone actions in cartilage and bone. Eur Thyroid J. 2013;2:3–13. 10.1159/000345548 PMC3821494 24783033

[43] Wopereis DM , Du Puy RS , van Heemst D , Walsh JP , Bremner A , Bakker S , et al. The relation between thyroid function and anemia: a pooled analysis of individual participant data. J Clin Endocrinol Metab. 2018;103:3658–3667. 10.1210/jc.2018-00481 PMC6179176 30113667

[44] Workgroup of the American Association of Hip and Knee Surgeons Evidence Based Committee . Obesity and total joint arthroplasty: a literature based review. J Arthroplasty. 2013;28:714–721. 10.1016/j.arth.2013.02.011 23518425

[45] Wyne KL , Nair L , Schneiderman CP , Pinsky B , Antunez Flores O , Guo D , et al. Hypothyroidism prevalence in the United States: a retrospective study combining National Health and Nutrition Examination Survey and Claims Data, 2009‐2019. J Endocr Soc. 2022;7:bvac172. 10.1210/jendso/bvac172 PMC9706417 36466005

[46] Yang Q , Wang J , Xu Y , Chen Y , Lian Q , Zhang Y . Incidence and risk factors of in‐hospital prosthesis‐related complications following total hip arthroplasty: a retrospective Nationwide Inpatient Sample database study. Int Orthop. 2020;44:2243–2252. 10.1007/s00264-020-04682-y 32594223

[47] Yu S , Garvin KL , Healy WL , Pellegrini VD , Iorio R . Preventing hospital readmissions and limiting the complications associated with total joint arthroplasty. Instr Course Lect. 2016;65:199–210. 27049191

