# Supplementary material for: Hypothyroidism is associated with higher healthcare utilisation and higher need for blood transfusion after primary total knee arthroplasty (TKA) in patients with osteoarthritis: A National Inpatient Sample analysis
Source: J Exp Orthop. 2026 Jan 30;13(1):e70413. doi: 10.1002/jeo2.70413 (PMC12856714; doi:10.1002/jeo2.70413)
Supplement: Supplementary file 1 — Supporting information. [file JEO2-13-e70413-s001.docx]

**Figure S1.** Time-trends in the underlying diagnosis for primary TKA among people with and without hypothyroidism


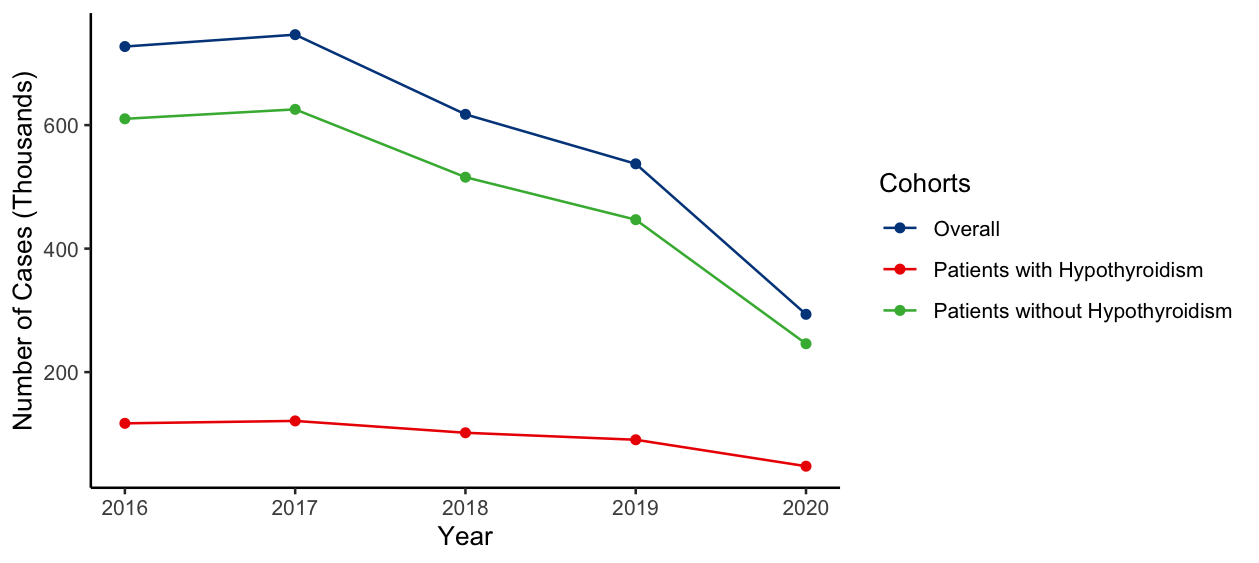


**Figure Legend.** The overall number of total primary TKA with underlying diagnosis of osteoarthritis decreased between 2016-2020, with a more drastic decrease in 2020, as expected during the COVID-19 pandemic, early phase. The overall number of people without hypothyroidism significantly decreased as well, while the overall number of patients with hypothyroidism stayed relatively constant prior to 2020.

**Figure S2.** Adjusted Odds ratio of significant association of hypothyroidism with select clinical and healthcare utilization outcomes of total TKA population


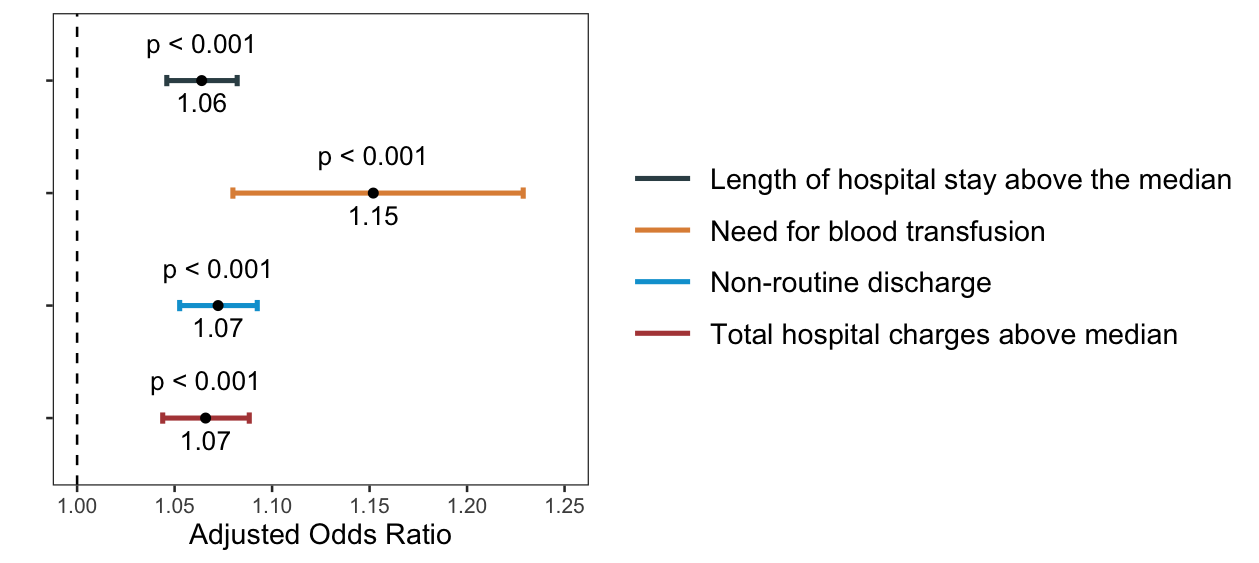


**Figure Legend.** The adjusted odds ratios are from the logistic regression model, including the entire TKA population with an underlying diagnosis of OA, that assessed the association of hypothyroidism with poor clinical and healthcare utilization outcome using the NIS data from 2016 to 2020. Only associations that were statistically significant with p-value <0.05 are shown. The point estimates are the adjusted odds ratios, and the whiskers represent the 95% confidence intervals. The null hypothesis is represented by the dotted line crossing the odds ratio of 1.

**Table S1. Underlying conditions and post-operative complications of interest and the corresponding ICD-10 codes**

| **ICD-10 Code** | **Description** |
| --- | --- |
| **Underlying Condition Codes** | |
| Osteoarthritis | |
| M17 | Osteoarthritis of knee |
| **Post-Operative Complication Codes for the index primary TKA** | |
| Blood Transfusion (Procedure code): | |
| 30233N1 | Transfusion of Non-autologous Red Blood Cells into Peripheral Vein, Percutaneous Approach |
| Prosthetic Fracture: | |
| T84012A | Broken internal right knee prosthesis, initial encounter |
| T84013A | Broken internal left knee prosthesis, initial encounter |
| T84018A | Broken internal joint prosthesis, other site, initial encounter |
| T84019A | Broken internal joint prosthesis, unspecified site, initial encounter |
| M9665 | Intraoperative and postprocedural complications and disorders of musculoskeletal system, not elsewhere classified |
| M96661 | Fracture of femur following insertion of orthopedic implant, joint prosthesis, or bone plate, right leg |
| M96662 | Fracture of femur following insertion of orthopedic implant, joint prosthesis, or bone plate, left leg |
| M96669 | Fracture of femur following insertion of orthopedic implant, joint prosthesis, or bone plate, unspecified leg |
| M9711XA | Periprosthetic fracture around internal prosthetic right knee joint, initial encounter |
| M9712XA | Periprosthetic fracture around internal prosthetic left knee joint, initial encounter |
| Prosthetic Dislocation: | |
| T84022A | Dislocation of internal right knee prosthesis, initial encounter |
| T84023A | Dislocation of internal left knee prosthesis, initial encounter |
| T84028A | Dislocation of other internal joint prosthesis, initial encounter |
| T84029A | Dislocation of unspecified internal joint prosthesis, initial encounter |
| **Post Procedural Infection:** | |
| Deep Surgical Site Infection (SSI) | |
| T8142XA | Infection following a procedure, deep incisional surgical site, initial encounter |
| Postprocedural sepsis | |
| T8144XA | Sepsis following a procedure, initial encounter |
| Periprosthetic Joint Infection | |
| T8450XA | Infection and inflammatory reaction due to unspecified internal joint, initial encounter |
| T8453XA | Infection and inflammatory reaction due to internal right knee prosthesis, initial encounter |
| T8454XA | Infection and inflammatory reaction due to internal left knee prosthesis, initial encounter |
| T8459XA | Infection and inflammatory reaction due to other internal joint prosthesis, initial encounter |

**Table S2. Characteristics of patients undergoing primary TKA 2016-2020, entire cohort vs. those with a primary diagnosis of osteoarthritis**

|  | Patients **with** primary TKA, all causes (N= 2,968,580; 100%) | **All** **patients** with osteoarthritis (N= 2,922,075; 98.4%) |
| --- | --- | --- |
| Age in years, mean (SD) | 68.7 (12.3) | 66.8 (9.4) |
| Sex, N (%) | | |
| Male | 1,142,635 (38.5) | 1,126,475 (38.6) |
| Female | 1,825,385 (61.5) | 1,795,070 (61.4) |
| Race/Ethnicity, N (%) | | |
| White | 2,320,060 (81.3) | 2,285,875 (81.3) |
| Black | 232,115 (8.1) | 227,870 (8.1) |
| Hispanic | 180,205 (6.3) | 176,640 (6.3) |
| Asian or Pacific Islander | 44,900 (1.6) | 44,240 (1.6) |
| Other | 77,910 (2.7) | 76,700 (2.7) |
| Deyo-Charlson Comorbidity Index, N (%) | | |
| 0 | 1,675,660 (56.4) | 1,656,465 (56.7) |
| 1 | 747,345 (28.6) | 833,280 (28.5) |
| ≥2 | 444,575 (15.0) | 432,330 (14.8) |
| Insurance Type, N (%) | | |
| Medicare | 1,687,455 (56.9) | 1,661,585 (56.9) |
| Medicaid | 127,790 (4.3) | 125,490 (4.3) |
| Private insurance, self-pay, no charge, or other | 1,148,660 (38.7) | 1,131,400 (38.8) |
| Median Household Income for ZIP Code, N (%) | | |
| 0-25th percentile | 646,425 (22.1) | 635,140 (22.0) |
| 26th-50th percentile | 781,445 (26.7) | 768,825 (26.7) |
| 51st to 75th percentile | 792,440 (27.1) | 780,715 (27.1) |
| 76th to 100th percentile | 706,475 (24.1) | 696,370 (24.2) |
| Hospital Bed size, N (%) | | |
| Small | 968,430 (32.6) | 953,605 (32.6) |
| Medium | 836,054 (28.2) | 823,569 (28.2) |
| Large | 1,164,095 (39.2) | 1,144,900 (39.2) |
| Census Division of Hospital, N (%) | | |
| Northeast | 566,511 (19.1) | 559,676 (19.2) |
| Midwest | 772,156 (26.0) | 759,041 (26.0) |
| South | 1,061,200 (35.7) | 1,043,850 (35.7) |
| West | 568,714 (19.2) | 559,509 (19.1) |
| Location/teaching status of hospital, N (%) | | |
| Rural | 309,210 (10.4) | 304,520 (10.4) |
| Urban nonteaching | 804,136 (27.1) | 791,266 (27.1) |
| Urban teaching | 1,855,234 (62.5) | 1,826,289 (62.5) |
| Emergency service | | |
| No emergency department indicator | 2,942,195 (99.1) | 2,900,895 (99.3) |
| Emergency department record | 26,385 (0.9) | 21,180 (0.7) |
| ^1^includes the presence of one or more of the following conditions based on the respective ICD-10 codes: rheumatoid arthritis, spondyloarthritis, ankylosing spondylitis and/or psoriatic arthritis | | |

**Table S3. Time-trends in all Post-primary TKA outcomes with an underlying diagnosis of OA from 2016-2020 for overall cohort**

|  | All Patients | Study Time Periods | | | | | Comparison of 2019 to 2016 | |
| --- | --- | --- | --- | --- | --- | --- | --- | --- |
|  | 2016-2020 | 2016 | 2017 | 2018 | 2019 | 2020 | Last–first period (% difference) | p-value |
| Length of hospital stay in days, median (IQR) | 2 (1-3) | 2 (2-3) | 2 (1-3) | 2 (1-3) | 2 (1-3) | 2 (1-2) | 0.0 | ***** |
| Total hospital charges in $, median (IQR) | 52,308.0 (38,062.0-74,500.0) | 50,883.5 (37,156.3-71,124.8) | 51,365.0 (37,691.0-72,790.8) | 52,524.0 (38,255.0-75,492.0) | 53,686.0 (38,812.5-77,166.3) | 55,593 (39,541.0-81,242.0) | **5.5** | **<0.001** |
| Inpatient mortality, N (%) | 695 (0.02) | 150 (0.02) | 185 (0.02) | 150 (0.02) | 115 (0.02) | 95 (0.03) | 3.7 | 0.60 |
| Non-routine discharge, N (%) | 1,807,010 (61.9) | 471,425 (64.9) | 467,370 (62.7) | 377,644 (61.2) | 320,515 (59.7) | 170,055 (57.9) | **-8.1** | **<0.001** |
| **Complications (Initial Visit), N (%)** | | | | | | | | |
| Need for blood transfusion | 32,110 (1.1) | 11,175 (1.5) | 8,180 (1.1) | 6,045 (1.0) | 4,270 (0.8) | 2,440 (0.8) | **-48.3** | **<0.001** |
| Prosthetic fracture | 3,075 (0.1) | 760 (0.1) | 790 (0.1) | 575 (0.1) | 600 (0.1) | 350 (0.1) | 6.9 | 0.54 |
| Prosthetic dislocation | 550 (0.02) | 135 (0.02) | 100 (0.01) | 115 (0.02) | 100 (0.02) | 100 (0.03) | 0.30 | 0.05 |
| Post-procedural Infection^1^ | 320 (0.01) | 50 (0.01) | 75 (0.01) | 80 (0.01) | 75 (0.01) | 40 (0.01) | 103 | 0.39 |
| Peri-prosthetic joint infection | 225 (0.01) | 50 (0.01) | 75 (0.01) | 50 (0.01) | 30 (0.01) | 20 (0.01) | -18.8 | 0.75 |
| ^1^ significant p-values are bolded | | | | | | | | |
| *Data excluded due to HCUP guidelines | | | | | | | | |
| **Not assessed since there was no difference between 2016 to 2019 | | | | | | | | |

**Table S4. Utilization Rates for primary TKA with an underlying diagnosis of OA for patients with hypothyroidism**

| Year | Number of patients with OA with hypothyroidism who underwent primary TKA | Total number of all patients underwent primary TKA | Total number of primary TKA for OA | % of OA and TKA Population | P-value based on the Cochran Armitage Test^1^ |
| --- | --- | --- | --- | --- | --- |
| 2016 | 117,050 | 739,581 | 727,251 | 16.09 | **<0.001** |
| 2017 | 120,965 | 757,249 | 746,464 | 16.21 |  |
| 2018 | 101,770 | 626,289 | 617,454 | 16.48 |  |
| 2019 | 90,455 | 545,541 | 537,311 | 16.83 |  |
| 2020 | 47,635 | 299,921 | 293,596 | 16.22 | * |
| ^1^ Cochran Armitage test compares the number of patients with OA with hypothyroidism who underwent primary TKA from 2016-2019 | | | | | |
